# Concurrent anthropogenic stressors affect plant–soil systems with different plant diversity levels

**DOI:** 10.1111/nph.70275

**Published:** 2025-06-04

**Authors:** Yanjie Zhu, Peter Meidl, Huiying Li, Mohan Bi, Masahiro Ryo, Matthias C. Rillig

**Affiliations:** ^1^ Institute of Biology Freie Universität Berlin 14195 Berlin Germany; ^2^ Berlin‐Brandenburg Institute of Advanced Biodiversity Research (BBIB) 14195 Berlin Germany; ^3^ Leibniz Centre for Agricultural Landscape Research (ZALF) 15374 Müncheberg Germany; ^4^ Brandenburg University of Technology Cottbus–Senftenberg (BTU‐CS) 03046 Cottbus Germany

**Keywords:** biodiversity, ecosystem functioning, global change, interactive effect, plant community, plant functional group evenness, sampling effect

## Abstract

- Plant diversity strongly influences ecosystem functioning. Due to human activities, ecosystems are increasingly threatened by the co‐occurrence of numerous anthropogenic pressures, but how they respond to this multifaceted phenomenon is poorly documented, and what role plant diversity plays in this process has not been investigated so far.
- Here, plant–soil systems with different plant diversity levels (3 vs 9 species) were subjected to an increasing number of anthropogenic stressors (0, 1, 2, 5, and 8).
- Results show that soil properties and functions were directionally driven by stressor number, irrespective of plant diversity level, and plant functional group evenness declined continuously along the stressor number gradient. The impact of stressors on plant–soil systems varied depending on plant diversity, and when plant diversity was higher, concurrent stressors may have interacted more to affect plant–soil systems. Notably, increasing the stressor number tended to diminish the effects of plant diversity.
- This study represents a first attempt to address the effect of plant diversity under multi‐stressor combinations and highlights the importance of emphasizing plant–soil systems in the research field of multifactorial global change. We also suggest that efforts should be made to reduce the number of coacting stressors when managing plant–soil ecosystems.

## Introduction

Soils underpin a wide range of essential ecosystem functions and services, while global environmental change driven by anthropogenic activities is progressively threatening soil health (Barnosky *et al*., 2012; Oliver *et al*., 2015; Zandalinas *et al*., 2024). After several decades of being limited to single or double stressors (Rillig *et al*., 2019), research has recently begun to embrace the simultaneous action of multiple global change stressors such as climate change and chemical pollution (Zandalinas & Mittler, 2022). A few studies reported the emergence of detrimental effects with an increasing number of stressors, for example, inhibiting nutrient cycling, destabilizing soil aggregates, reducing microbial diversity, and plant productivity (Komatsu *et al*., 2019; Rillig *et al*., 2019; Zandalinas *et al*., 2021; Speißer *et al*., 2022; Yang *et al*., 2022; Sinha *et al*., 2023; Bi *et al*., 2024; Liu *et al*., 2024), emphasizing the need for global change research to focus on the joint effects of numerous stressors.

When multiple stressors act together, the joint impact can simply be additive, equivalent to the sum of their independent effects, or they may interact with each other either synergistically or antagonistically. Whether and how stressors interact usually depends on treatment length, intensity, and the type of function considered (Zhou *et al*., 2016; Ma *et al*., 2020). The mechanisms behind such interactions are complicated; one factor can directly influence another before affecting the target system, while they may also interact indirectly via the medium of plants and soil biota (Rillig *et al*., 2021a; Zandalinas *et al*., 2024). With the increase in the number of co‐acting anthropogenic stressors (hereafter stressor number), there may be unforeseen consequences due to factor interactions, and higher uncertainty in predicting the responses of ecosystems (Rillig *et al*., 2019; Zhou *et al*., 2023). Occasionally, although the individual effects of single stressors are negligible, their joint effect can be unexpectedly strong (Rillig *et al*., 2019; Zandalinas *et al*., 2021; Speißer *et al*., 2022; Bi *et al*., 2024). As such, identifying factor interactions, especially high‐order interactions, is essential in multifactorial global change studies (Rillig *et al*., 2021a).

Plant–soil systems are critical for deciphering the ecological impacts of global change (Rillig *et al*., 2021a), yet how they are affected by a large number (e.g. up to 6) of concurrent stressors has rarely been investigated experimentally. Studies have targeted single plant species and found strong combined effects of multiple stressors on plant health (Zandalinas *et al*., 2021; Sinha *et al*., 2023; Peláez‐Vico *et al*., 2024), while one study scaled up to the community level, showing directional shifts in plant community structure following the increase of stressor number (Speißer *et al*., 2022). Within a plant community, plant diversity in terms of species number is considered crucial for the performance of plant–soil systems (Lange *et al*., 2015), and its positive contributions to ecosystem resistance and resilience under disturbed conditions have been frequently reported (Isbell *et al*., 2015). For example, plant diversity stabilizes the productivity of grassland communities because biomass decreases in some species can be compensated for by increases in other species (Tilman *et al*., 2006; Hector *et al*., 2010). Diverse plant communities provide varied inputs of litter and root exudates, which enhance the resistance of microbial communities (Li *et al*., 2022). The diverse root types may improve soil aggregate stability as well (Pohl *et al*., 2009). Soil temperature change is also found to be buffered by plant diversity, through increased soil organic carbon concentrations and plant leaf area index (Huang *et al*., 2024).

The relationship between plant diversity and ecosystem functioning is likely to be affected by global change (de Gea *et al*., 2023). Anthropogenic disturbances such as nutrient enrichment and drought were shown to jeopardize the favorable effects of plant diversity (Hautier *et al*., 2014, 2020; Hou *et al*., 2024). However, there is currently a complete lack of knowledge on how plant diversity mediates the response of plant–soil systems to multiple concurrent stressors of global change, and how stressor number shapes the relationship between plant diversity and the functioning of plant–soil ecosystems. To address these gaps, we conducted a mesocosm experiment in the controlled environment room, where an increasing number of stressors (0, 1, 2, 5, and 8) were imposed on grassland communities with low and high plant diversity (3 vs 9 species from three plant functional groups). A major challenge for such a multifactorial study is the combinatorial explosion problem; that is, when the stressor number increases, the number of possible combinations increases rapidly (Rillig *et al*., 2021b). Taking inspiration from the design of biodiversity experiments (Tilman *et al*., 1997), Rillig *et al*. (2019) proposed a method that creates a stressor number gradient using random assemblages of stressors drawn from a predefined factor pool. This design circumvents the combinatorial explosion problem without losing the generalizability of the study. Following this approach, we first assembled a stressor pool and a plant species pool, and then randomly selected stressors and plants from the pools for each experimental unit to achieve the simultaneous manipulation of plant diversity and stressor number.

The plant–soil mesocosms were maintained for 4 months, after which a series of response variables were measured, including soil respiration, litter decomposition, extracellular enzyme activities, water‐stable aggregates (WSAs), pH, overall root mass, and shoot mass of each plant species, to examine changes in soil properties, functions, and plant community composition. We hypothesized that (1) increasing the number of anthropogenic stressors will cause directional changes in these response variables, (2) plant diversity helps maintain ecosystem functions under multifactorial stress combinations, and (3) the co‐occurrence of multiple stressors eliminates the effects of plant diversity on ecosystem functions.

## Materials and Methods

### Test soil

The test soil (loamy sandy; Albic Luvisol) was collected from a grassland site (52°47′N, 13°30′E) belonging to the Institute of Biology, Freie Universität Berlin. The soil was air‐dried and passed through a 2‐mm sieve, and its basic physicochemical properties were measured (water holding capacity (WHC) 38.5%; pH 5.8). The air‐dried soil was homogeneously mixed with sterilized (autoclaved for 90 min at 121°C) sand (w/w = 1 : 1) in order to improve drainage, aeration, and avoid soil compaction, thus creating a more favorable environment for root growth. The WHC of the soil–sand mixture was 28.1%.

### Anthropogenic stressors

We defined a stressor pool containing eight stressors, including insecticide (imidacloprid, 50 μg kg^−1^), fungicide (carbendazim, 6.0 mg kg^−1^), antibiotic (oxytetracycline, 3.0 mg kg^−1^), heavy metal (copper, 100 mg kg^−1^), perfluoroalkyl and polyfluoroalkyl substances (perfluorooctanoic acid, 1 mg kg^−1^), surfactant (sodium dodecylbenzenesulfonate, 16 mg kg^−1^), microplastics (tire wear particles, 1 g kg^−1^), and drought (30% of WHC compared to 60%). This stressor pool was mainly composed of chemicals, especially synthetic chemicals, because they have been regarded as agents of global change (Bernhardt *et al*., 2017). It was reported that the quantity and diversity of synthetic chemicals released into the environment have been increasing at rates greatly surpassing those of other well‐recognized global change drivers, but they have been largely overlooked in global change studies (Bernhardt *et al*., 2017). The stressors we chose frequently co‐occur and pose threats to agroecosystems and urban soils (Yang *et al*., 2022). They differ in terms of their effects on soil, and thus can represent a relatively broad range of common global change factors (Rillig *et al*., 2021b; Bi *et al*., 2024). The rationale for each stressor is presented in Supporting Information Methods S1.

### Plants

The plant pool contained 12 species prevalent in Central European grasslands. Considering that plants from different functional groups may have distinct roles in affecting ecosystem functioning, we categorized these plant species into three functional groups, including four species of grasses (*Phleum pratense* L., *Poa annua* L., *Lolium perenne* L., *Dactylis glomerata* L.), four species of herbs (*Achillea millefolium* L., *Hypericum perforatum* L., *Daucus carota* L., *Rumex acetosella* L.), and four species of legumes (*Trifolium repens* L., *Trifolium pratense* L., *Medicago lupulina* L., *Lotus corniculatus* L.). The seeds of *A. millefolium* and *H. perforatum* were purchased from Jelitto Staudensamen GmbH (Schwarmstedt, Germany), and other species from Templiner Kräutergarten (Templin, Germany). Plant seeds were surface sterilized with 70% ethanol for 2 min, germinated in sterilized sand at room temperature, and hydrated with sterilized water. Germination started at different times according to the length of time required for each plant species to germinate (obtained from a preliminary test) to ensure their synchronous germination.

### Experimental design

In the experimental design, we carried out a constrained two‐dimensional random draw: (1) the random draw of stressors from the stressor pool; and (2) the random draw of plants from the plant pool. First, for each plant diversity level, a gradient of stressor number was created, namely factor levels 0, 1, 2, 5, and 8. Level 0 represents the control without the application of stressors, which was replicated 10 times. For level 1 (single‐stressor level), each of the eight stressors was replicated 6 times. Levels 2 and 5 were replicated 20 times respectively, and each time the corresponding numbers of stressors were randomly selected from the factor pool (i.e. ‘heterogeneous replicates’ (Clark *et al*., 2019)). Level 8 was also replicated 20 times, with all the stressors being applied, and each time, the plants were randomly selected from the plant pool. In total, there were (10 + 6 × 8 + 20 + 20 + 20) = 118 random assemblages of stressors. Then, each of the assemblages was combined with low and high plant diversity treatments, respectively, yielding 236 experimental units in total (Table 1). This design ensured that the randomly determined stressor composition was consistent in every pair of low and high plant diversity units so that the probability of detecting any plant species richness effects could be maximized. To be more specific, for the low plant diversity treatment, we randomly chose one plant species from each of the three functional groups and planted three seedlings for each species. For the high plant diversity treatment, we randomly chose three different plant species from each of the functional groups and planted one seedling for each species. In this way, the plant functional group richness was kept identical across all treatments.

**Table 1:** Experimental design: for each plant diversity level, there were 10 replicates for the control (stressor level 0), 6 replicates for each single stressor treatment (stressor level 1), and 20 replicates for each multiple‐stressors treatment (stressor levels 2, 5, and 8); total experimental units = (10 + 6 × 8 + 20 + 20 + 20) × 2 = 236.

Stressor level	Plant diversity	Replicates (*n*)
0	Low	10
1	Low	6 × 8 = 48
2	Low	20
5	Low	20
8	Low	20
0	High	10
1	High	6 × 8 = 48
2	High	20
5	High	20
8	High	20

### Experiment establishment and harvest

The plant–soil mesocosms were established in a controlled environment room (16 h : 8 h, 22°C : 18°C, light : dark (i.e. day : night)) with 236 pots (diameter, 16 cm; height, 16.5 cm). For each pot, we used 10.0 g of sterilized (autoclaved for 90 min at 121°C) ‘loading’ sand to achieve a more effective mixing of chemical agents into the soil and to avoid any exaggerated effects on the soil community (Rillig *et al*., 2019). The ‘loading’ sand was spiked with the appropriate dose of the chemical treatments, shaken thoroughly, homogenously mixed with 3 kg of soil–sand mixture, and then filled into the pot. A pre‐made litter bag filled with green tea was inserted vertically into the center of the pot for the determination of litter decomposition rate. The mesocosms were pre‐incubated at 60% WHC for a week to activate microbial communities (Erinle & Marschner, 2022). After the pre‐incubation, each pot was planted with the nine post‐germinated seedlings arranged in a 3 × 3 grid pattern, with their relative positions randomly assigned. All plants were well‐watered during the first 4 wk of growth. Starting from the fifth week after planting, mesocosms with the drought treatment were maintained at 30% WHC, while the others were kept at 60% WHC.

Ten weeks after planting, plant shoots were cut at *c*. 5 cm above the soil surface, simulating a mowing event typical of grassland, sorted by species, and oven‐dried at 60°C, after which all the mesocosms were incubated for six more weeks before the final harvest. In total, the experiment ran for 17 wk (1 wk of pre‐incubation and 16 wk of plant growth). Throughout the whole experimental period, all pots were watered two or three times a week, depending on their average water loss. Every 2 wk, each pot was weighed and watered precisely according to the accurate amount of water loss, and in the meantime, the locations of the pots in the controlled environment room were reassigned randomly.

At the final harvest, plant shoots were cut off completely and sorted by species, while roots were collected altogether without sorting. All the biomass was oven‐dried at 60°C. After the biomass harvest, the litter bags were collected, and each soil sample was carefully homogenized using a spatula. Then, 30.0 and 5.0 g of fresh soil samples were taken, respectively, and stored at 4°C for determining the soil respiration rate and extracellular enzyme activities. The rest of the soil samples were air‐dried and stored at room temperature.

### Response variables

The shoot mass of each plant species from the two harvests and the overall root mass of all plant species were weighed after oven‐drying. The total shoot mass of plants from each functional group was summed up. Plant functional group evenness in terms of plant yield was quantified by the Pielou evenness index (Pielou, 1969): $E_{\mathrm{FG}}=-\left(\sum_{i=1}^{F}G_{i}\times \log_{e}G_{i}\right)/\log_{e}F$, where *G*_*i*_ is the relative shoot mass of plant functional group *i* and *F* is the number of plant functional groups (Andruschkewitsch *et al*., 2014) (which was 3 across all treatments in this case). The calculation was performed by the R (v.4.4.0) package vegan (Oksanen *et al*., 2024).

We also measured variables that characterize soil microbial activities, including decomposition rate, soil respiration rate, and extracellular enzyme activities (measured within 2 wk after harvest), as well as physicochemical properties (water WSAs, pH). Briefly, the proportional loss of litter (green tea) dry weight in the litterbag during soil incubation was used to indicate the decomposition rate (Xie, 2020). The CO_2_ concentration produced by fresh soil per hour was analyzed by an infrared gas analyzer and used to indicate the soil respiration rate (Rillig *et al*., 2019). The activities of C‐related enzymes β‐glucosidase (EC3.2.1.21) and β‐d‐1,4‐cellobiosidase (EC3.2.1.91), N‐related enzyme β‐1,4‐N‐acetyl‐glucosaminidase (EC3.2.1.52), and P‐related enzyme phosphatase (EC3.1.3.2) were measured based on high‐throughput microplate assays, by exposing fresh soil to artificial *p*‐nitrophenyl (pNP) linked substrates and tracking the rate of substrate hydrolysis with a microplate reader (Jackson *et al*., 2013). WSAs were measured using the wet sieving method (Xu *et al*., 2022). Soil pH was determined by a pH meter. The detailed procedures and corresponding references for each measurement are presented in Methods S2.

### Assessing treatment effects

All the statistical analyses were performed in R (v.4.4.0), and the R packages ggplot2, ggepi, ggridges, patchwork, cowplot and ggpubr were used to visualize the results. First, the effect size of each single stressor was quantified by comparing the difference in means and 95% confidence intervals (CIs) between single factor treatments and control based on bootstrap resampling (5000 iterations) following the procedure from a previous study (Rillig *et al*., 2019). This nonparametric statistical method assesses the distribution of a statistic by random sampling with replacement from the original dataset repeatedly. It provides reliable estimates of mean and CIs without additional assumptions, and can reduce the undue influence of extreme data (Chernick, 2008; Kulesa *et al*., 2015). To be specific, for each bootstrap iteration, a new resampled dataset was generated by randomly drawing observations with replacement from the original treatment group and control group. Then, the mean was calculated for both groups in the resampled dataset, and the effect size was computed as the difference between these means. This procedure was repeated 5000 times, each time producing a new effect size estimate, resulting in a distribution of 5000 effect size values (the effect sizes of single stressors obtained here were also used for further null model analyses to predict the effect sizes of multi‐stressors). From these values, the mean effect size and its 95% CI was calculated. The *P*‐value was determined by calculating the proportion of bootstrap samples where the effect size was greater than zero, and then adjusting for a two‐tailed test.

The same bootstrap resampling method was applied to assess the effect of plant diversity by comparing the difference in means and 95% CI between low and high plant diversity treatments. The 5000 effect size values obtained here were used for further regression analyses between the effect of plant diversity on response variables and stressor number.

Then, generalized additive models were fitted using the ‘gam’ function in the mgcv package to estimate the relationships between response variables and stressor number, as well as the relationships between the effect of plant diversity on response variables and the stressor number. The number of basis functions to use for each smooth term was set to 4 after seeking for the best parameter while testing other values.

### Identifying types of interactions among stressors

In multifactorial studies, null models are often used to predict the combined effects of multiple stressors by the effects of single stressors without considering factor interactions (Thompson *et al*., 2018a). A statistically significant deviation of the actual observation from the null prediction indicates synergistic or antagonistic interaction among stressors (Thompson *et al*., 2018a). In order to identify interaction types, we applied three null models (additive, multiplicative, and dominative models) for each sample from the two, five, or eight stressor treatments to predict the combined effect of stressors using the effect sizes (see ‘Assessing treatment effects’ in the Materials and Methods section) of the constituent single stressors (Schäfer & Piggott, 2018). The additive model assumes the joint effect of multiple stressors to be equivalent to the sum of their individual effects; the multiplicative model mathematically combines the proportional changes due to the effects of single stressors as if they acted consecutively; the dominative model assumes that the factor with the strongest effect overrides the others, thus the highest absolute value will be picked (Schäfer & Piggott, 2018). For details of the calculation, see Rillig *et al*. (2019).

The actual observations were then compared with the predictions from the three null models respectively. Observations within the 95% CIs of the null predictions were categorized as ‘no interaction’, while others were categorized depending on the type of response and direction. For variables with positive responses to stressors (compared to the control), observations that did not fit within the CIs of the null predictions were classified as ‘synergistic interaction’ when DN (the deviation from the null predictions) > 0 (i.e. observation − null prediction > 0), and as ‘antagonistic interaction’ when DN < 0. For variables with negative responses, they were classified as ‘synergistic interactions’ when DN < 0, and as ‘antagonistic interaction’ when DN > 0 (Bi *et al*., 2024).

### Evaluating the contributions of stressor number and plant diversity

As stressor number increases, there is a greater chance of incorporating stressors with particularly strong effects on soil properties and functions, resulting in an observed ‘stressor number effect’ that does not stem from stressor number *per se*, but is a result of the identity of the selected stressors (i.e. the so‐called ‘sampling effect’) (Loreau & Hector, 2001). To unravel effects, we applied a hierarchically‐nested model comparison approach following the framework proposed by Bi *et al*. (2024). Specifically, in the baseline model (Model 1), each response variable was modeled using the null predictions from the three null models. Then, ‘number of stressors’ (1, 2, 5, and 8) was added as an additional explanatory variable (Model 2), and afterwards, the variable ‘plant diversity’ (3 vs 9) was also included (Model 3). The three models were all generalized additive models (GAM) built by the ‘gam’ function in the mgcv package. The number of basis functions to use for each smooth term was set to 3 after seeking for the best parameter while testing other values. We then evaluated the performance of each model (i.e. how well each model explains the variability of the response variable, *R* ^2^) by the ‘summary’ function, and applied bootstrap resampling (5000 iterations) to estimate the 95% CI of *R* ^2^. ANOVA tests were performed by the ‘anova’ function to compare the model performance (Speißer *et al*., 2022). Since that null models assume the joint effect of multiple stressors to be exclusively attributed to the individual effects of single stressors, a great predictability of Model 1 indicates a strong ‘sampling effect’, and a statistically higher (*P* < 0.05) predictability of Model 2 compared to Model 1 suggests that the variability of the response variable is not solely due to stressor identity, but that stressor number itself also plays a role. Additionally, a higher predictability of Model 3 compared to Model 2 suggests that plant diversity is another factor affecting the response variable under multifactorial global change.

Considering that plant diversity may influence soil properties and functions directly or indirectly through its effects on biomass (Allan *et al*., 2011; Li *et al*., 2022), an additional analysis was performed with applied partial least squares path modeling (PLS‐PM), where the number of stressors and plant diversity were designated as exogenous variables, while plant shoot and root mass as mediators, and other response variables as endogenous variables. After standardizing the raw data using the ‘standardize’ function, models using the ‘plspm’ function in the plspm package with default parameters were constructed (Sanchez *et al*., 2015).

## Results

### Changes in the plant–soil systems under an increasing number of co‐occurring anthropogenic stressors

**Fig. 1:**
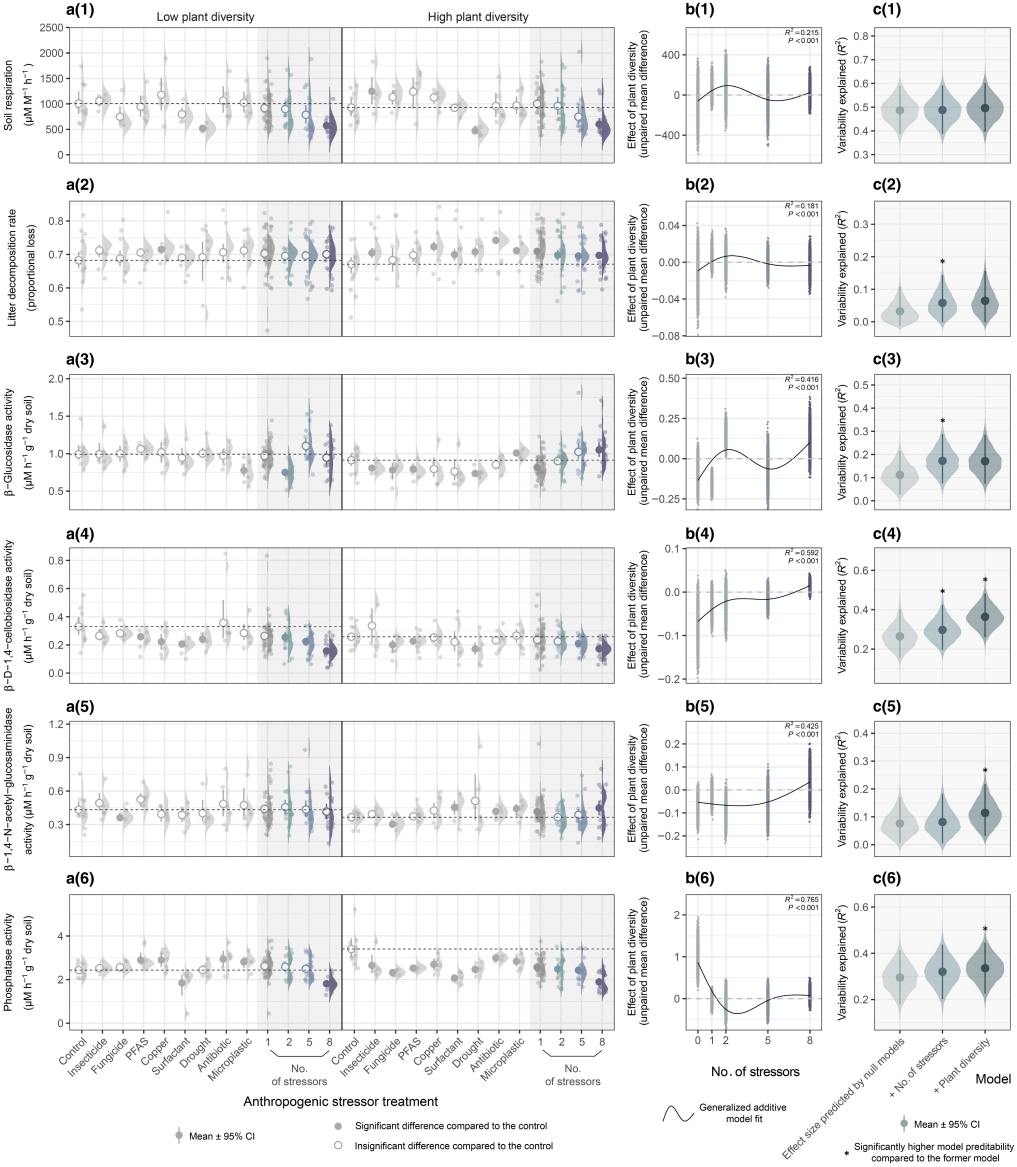
Soil microbial activities in response to different levels of anthropogenic stressors and plant diversity. (a(1)–a(6)) Raw data distribution, mean, and confidence interval (CI) of each response variable in treatments with single and multiple (2, 5, and 8) stressors under low and high plant diversity conditions. The filled circle represents a significant difference (*P* < 0.05) compared to the control, while the empty circle represents an insignificant difference (*P* > 0.05) compared to the control. The *P*‐values were calculated based on bootstrap resampling with 5000 iterations (Supporting Information Table S1). The horizontal dashed line represents the mean value of the control. (b(1)–b(6)) Correlations between the effect of plant diversity (unpaired mean difference between the low and high plant diversity treatments) on each response variable and the number of anthropogenic stressors. (c(1)–c(6)) Variability of response variable explained by generalized additive models. The baseline model using effect size predicted by null models represents the contribution of stressor identity, while added predictors represent the contributions of stressor number and plant diversity. The model comparisons were performed using ANOVA tests (Table S2).

**Fig. 2:**
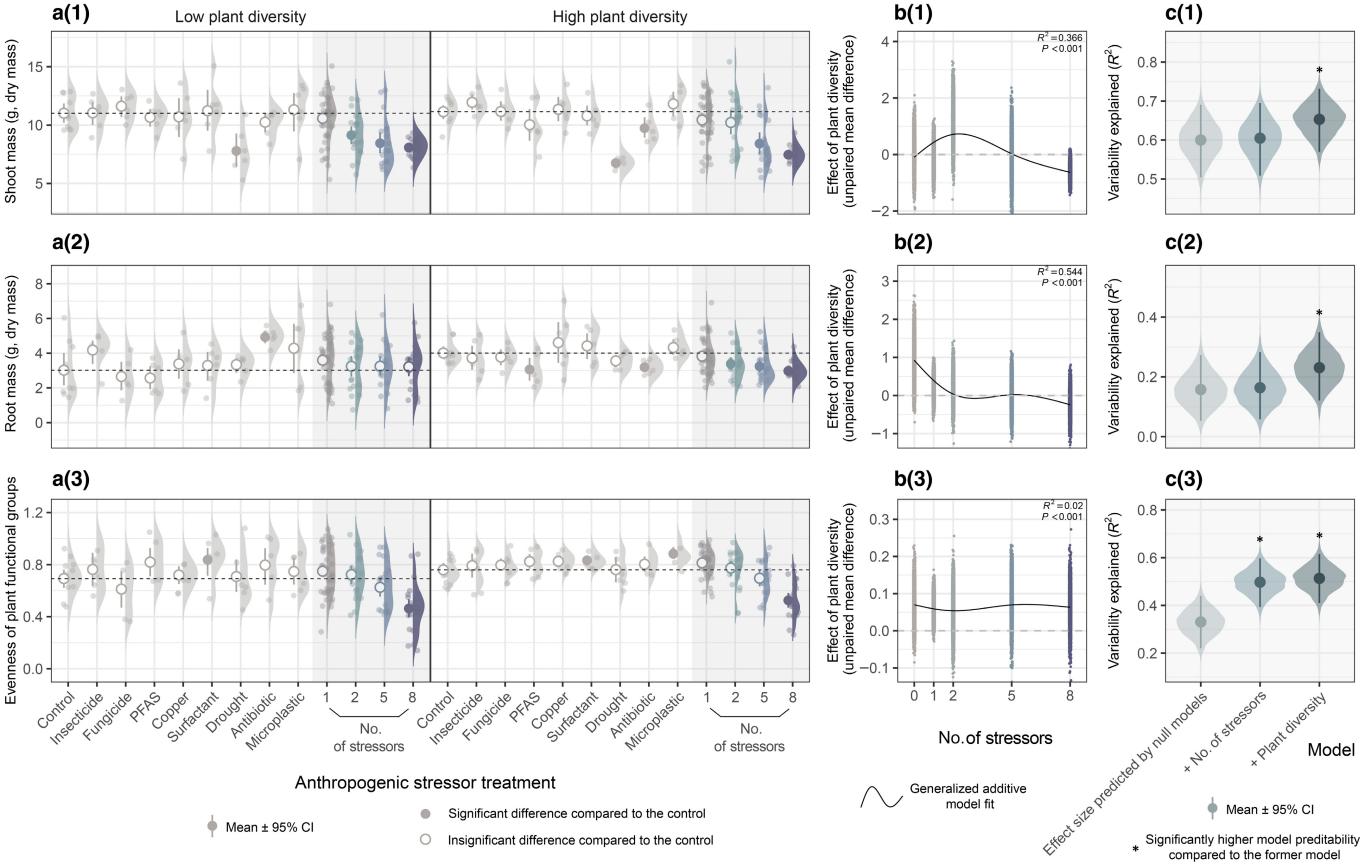
Overall plant shoot, root mass and functional group evenness in response to different levels of anthropogenic stressors and plant diversity. (a(1)–a(3)) Raw data distribution, mean, and confidence interval (CI) of each response variable in treatments with single and multiple (2, 5, and 8) stressors under low and high plant diversity conditions. The filled circle represents a significant difference (*P* < 0.05) compared to the control, while the empty circle represents an insignificant difference (*P* > 0.05) compared to the control. The *P*‐values were calculated based on bootstrap resampling with 5000 iterations (Supporting Information Table S1). The horizontal dashed line represents the mean value of the control. (b(1)–b(3)) Correlations between the effect of plant diversity (unpaired mean difference between the low and high plant diversity treatments) on each response variable and the number of anthropogenic stressors. (c(1)–c(3)) Variability of response variable explained by generalized additive models. The baseline model using effect size predicted by null models represents the contribution of stressor identity, while added predictors represent the contributions of stressor number and plant diversity. The model comparisons were performed using ANOVA tests (Table S2).

**Fig. 3:**
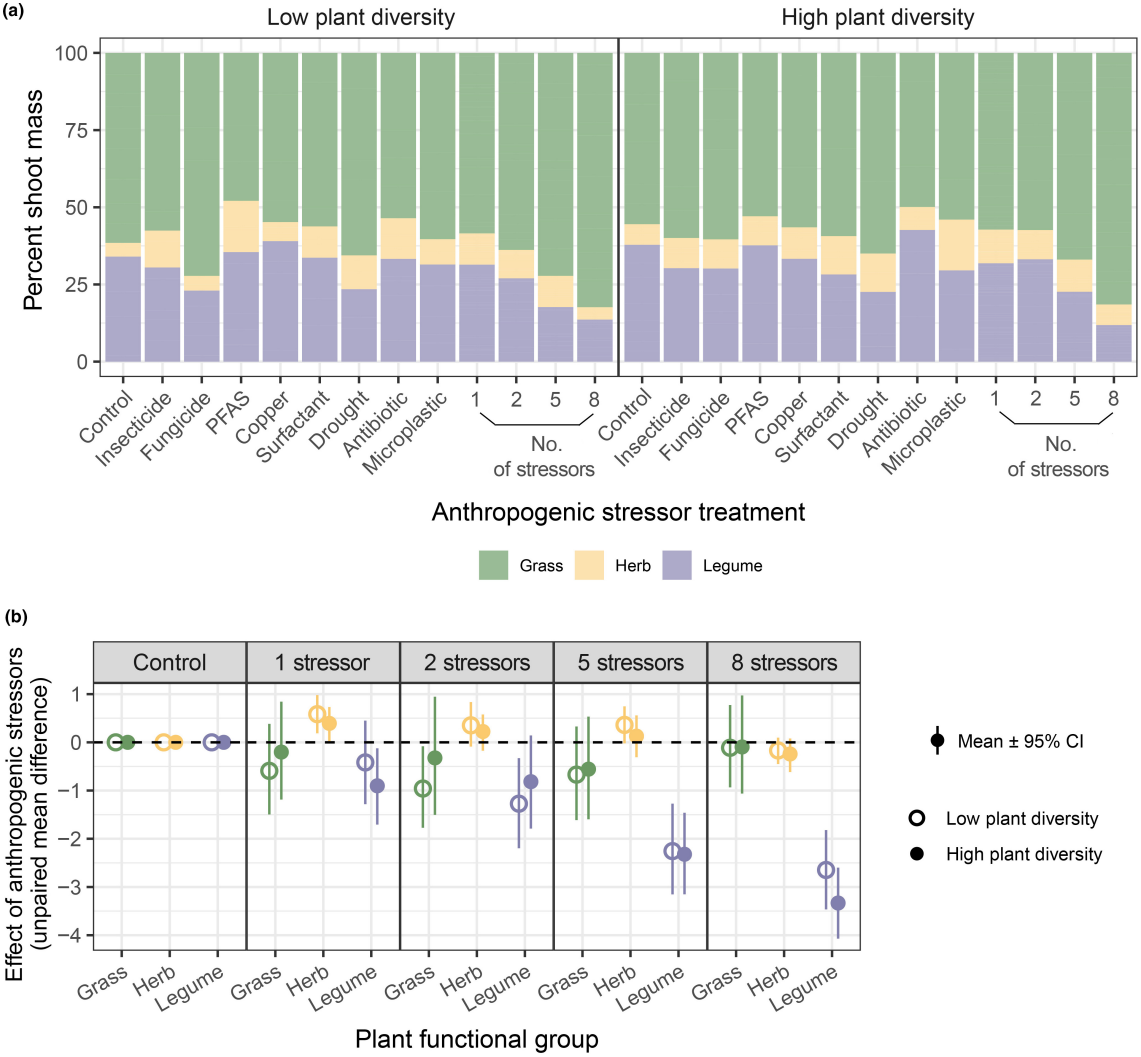
Effect of anthropogenic stressors on plant community composition in terms of functional group shoot mass. (a) Relative shoot mass of grasses, herbs, and legumes in different anthropogenic stressor treatments under low and high plant diversity conditions. (b) Effect of different numbers of stressors on shoot mass of grasses, herbs, and legumes under low and high plant diversity conditions.

In both low and high plant diversity systems, increasing the number of co‐acting anthropogenic stressors led to directional changes in soil properties, functions, and plant community composition (Fig. S2). For example, microbial activity indicators, including soil respiration, β‐d‐1,4‐cellobiosidase activity, and phosphatase activity, all exhibited downward trends (Fig. 1a(1),a(4),a(6)). Overall shoot mass was reduced significantly, while root mass was relatively stable (Fig. 2a(1),a(2)). Within the plant community, functional groups differed in their responses to the increasing number of stressors; the shoot mass of legumes declined pronouncedly, while there were only minor variations in herbs and grasses (Figs 3b, S1a(3)–a(5)), causing a decrease in functional group evenness (Fig. 2a(3)). Grasses have become increasingly dominant in the plant community with the increasing number of stressors (Fig. 3a).

The joint effect of multiple stressors can deviate far from the additive effects of single stressors, as evidenced by the elevated β‐glucosidase activity with an increasing number of stressors in high plant diversity systems, although single stressors mostly had adverse effects (Fig. 1a(3) right panel); such disparity also appeared on plant functional evenness in both low and high plant diversity systems, as single stressors barely affected, or only marginally increased plant functional group evenness, while it decreased continuously as factor number increased (Fig. 2a(3)).

### Effects of anthropogenic stressors under different plant diversity conditions

By comparing the effects of stressors on response variables in low and high plant diversity systems, we found that some stressors showed contrasting impacts depending on the plant diversity level (Fig. 1, column a; Table S1). For instance, most single stressors had no significant effect on litter decomposition in low plant diversity systems, while they had significant positive effects in high plant diversity systems. Such differences still existed when stressors were combined (Fig. 1a(2)). Microplastic reduced β‐glucosidase activity in low plant diversity systems, whereas the opposite was true in high plant diversity systems (Fig. 1a(3)). Except for microplastic, none of the single stressors significantly influenced β‐glucosidase activity in low plant diversity systems, while most of them elicited adverse effects in high plant diversity systems (Fig. 1a(3)), and the joint effects of two, five, and eight stressors also showed different tendencies under different plant diversity. Most single stressors had positive or insignificant effects on phosphate activity in low plant diversity systems, while all of them had negative effects in high plant diversity systems (Fig. 1a(4)). The root mass of the plant community was substantially elevated by antibiotic in low plant diversity systems, while reduced in high plant diversity systems (Fig. 1a(5)).

**Fig. 4:**
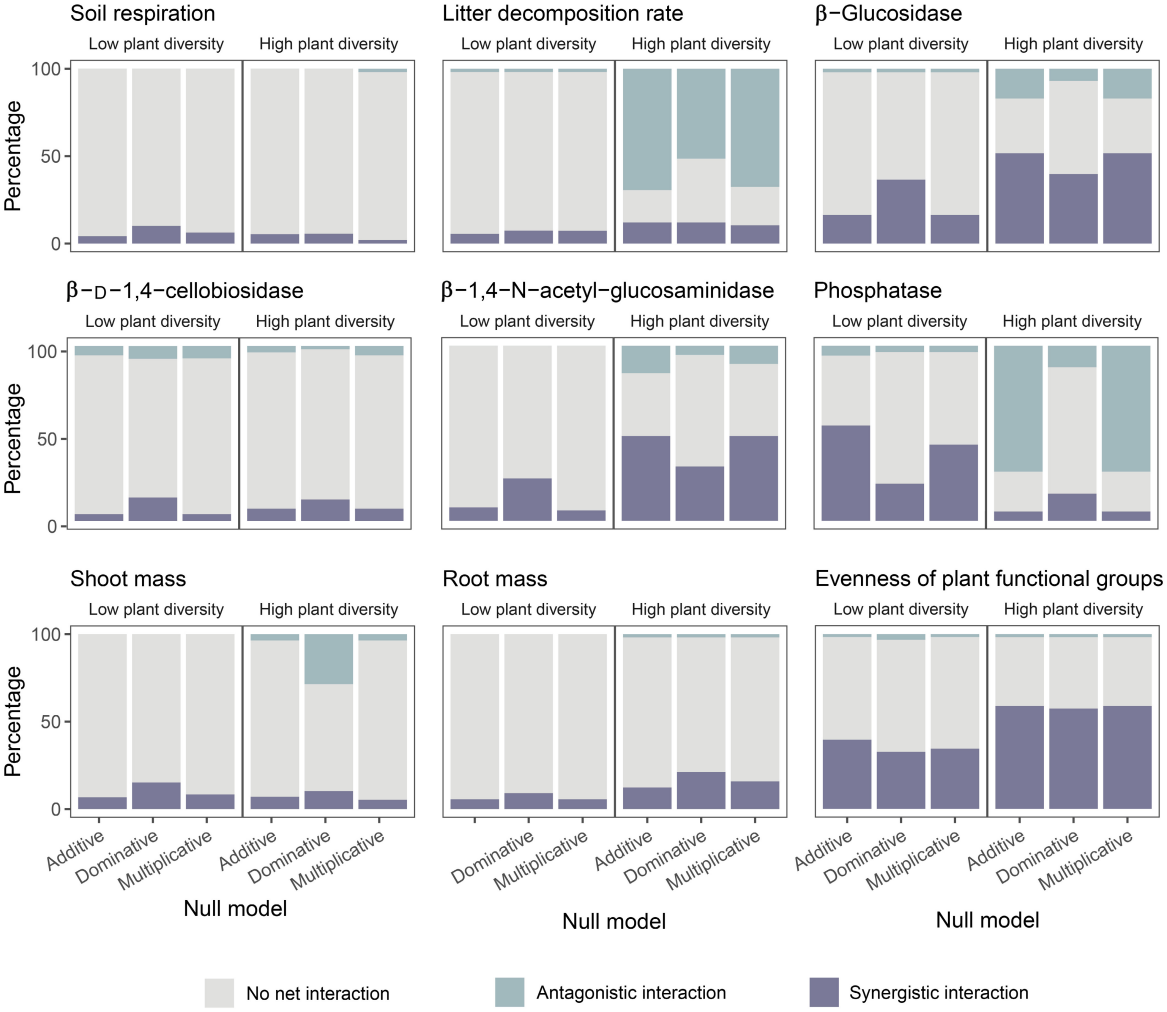
Percentage of different interaction types among concurrent anthropogenic stressors under low and high plant diversity conditions as identified by null models (additive, dominative, and multiplicative models). Interaction types (no net interaction, antagonistic, synergistic) are indicated by different colors.

Apart from discrepancies in the net effects observed, whether and how stressors interact to jointly influence plant–soil systems also differed depending on plant diversity. By comparing the actual observations with the null predictions, we found that in low plant diversity systems, whether based on additive, multiplicative or dominative null models, stressors did not interact to affect litter decomposition in > 90% of the cases, while in high plant diversity systems, ‘antagonistic interaction’ accounted for *c*. 50–70% of the interaction types. Analogously, for other response variables including β‐glucosidase, β‐1,4‐N‐acetyl‐glucosaminidase, phosphatase, shoot mass and plant functional group evenness, there appeared to be more stressor interactions under high plant diversity conditions, whereas ‘no net interaction’ always prevailed over antagonistic and synergistic interactions under low plant diversity conditions (Fig. 4).

### Relationships between the effects of plant diversity and the number of anthropogenic stressors

Plant diversity could either neutrally, negatively, or positively affect soil properties, functions, and plant community composition. We observed, on average, higher phosphatase activity, root mass, plant functional group evenness, and shoot mass of herbs and legumes (Figs 1b(4), 2b(2),b(3), S1a(4),a(5)), as well as lower β‐d‐1,4‐cellobiosidase activity, β‐1,4‐N‐acetyl‐glucosaminidase activity, WSA, and grass shoot mass (Figs 1b(3)–b(5), S1a(3)) in the high plant diversity systems compared to the low plant diversity systems, and these differences were usually more evident when fewer stressors were imposed. Increasing the number of stressors tended to minimize the differences between the low and high plant diversity systems. However, plant functional group evenness was an exception to this pattern; irrespective of the stressor number imposed, plant functional group evenness was always higher in high plant diversity systems than in the low counterpart (Fig. 2b(3)).

### Contributions of stressor number and plant diversity to changes in plant–soil systems

Hierarchically nested model comparison reflected the contributions of stressor number and plant diversity to the variations of soil properties, functions and plant community composition. Based on the effect size predicted by null models, adding the variable ‘number of stressors’ significantly increased model predictability (*R* ^2^) on litter decomposition, β‐glucosidase activity, β‐d‐1,4‐cellobiosidase activity, soil pH, legume shoot mass, especially on plant functional group evenness, which increased by 16.67% (Figs 1c(2)–c(4), 2c(3), S1c(2),c(5); Table S2). Moreover, for nearly 80% of the response variables (11 out of 14, all the measured variables included), adding ‘plant diversity’ as another explanatory variable further increased model predictability significantly (*R* ^2^) on nearly 80% of the response variables (Table S2).

Plant diversity can either directly affect soil properties and functions, or indirectly via changing plant biomass. However, the results of PLS‐PM showed that indirect effects mediated by plant shoot and root mass generally accounted for only a minor proportion of the total effects of plant diversity. For example, plant diversity affected β‐d‐1,4‐cellobiosidase activity directly with an estimated coefficient of −0.14851, while indirectly with an estimated coefficient of 0.00225; it affected β‐1,4‐N‐acetyl‐glucosaminidase activity directly with an estimated coefficient of −0.1269, while indirectly with an estimated coefficient of 0.00237 (Table S3).

## Discussion

In the present study, the effects of an increasing number of co‐occurring anthropogenic stressors on plant–soil systems with low and high plant diversity have been explored experimentally. In agreement with earlier studies (Komatsu *et al*., 2019; Rillig *et al*., 2019; Zandalinas *et al*., 2021; Speißer *et al*., 2022; Yang *et al*., 2022; Sinha *et al*., 2023; Bi *et al*., 2024; Liu *et al*., 2024), our results reveal that soil properties and functions were directionally driven by stressor number, despite plant diversity level. In addition to this, we also showed a notable decrease in plant functional evenness with increasing stressor number, even if the independent effects of single stressors were minor. Plant diversity affected not only the effects of stressors, but also their interactions, as stressors interacted more to affect plant–soil systems when plant diversity was higher. However, the effects of plant diversity tended to diminish as the number of stressors increased.

Soil respiration, β‐d‐1,4‐cellobiosidase activity, phosphatase activity, overall shoot mass, legume shoot mass, and plant functional group evenness exhibited clear decreasing trends along the stressor number gradient under both plant diversity conditions (Fig. S2). These alterations may originate from the identities of the selected stressors, the stressor number itself, or both. The hierarchically nested model comparison provided a general understanding of their respective contributions to the observed variations. For example, for soil respiration and overall shoot mass, drought was a stressor with particularly strong negative effects (Figs 1a(1), 2a(1)). With the increase of stressor number, the chance of including drought in the stressor combinations would also increase (sampling effect). According to the model comparison, null models explained nearly 50% of the variability in soil respiration and 60% of the variability in overall shoot mass, indicating strong sampling effects, while based on these null models, adding the explanatory variable ‘number of stressors’ did not significantly increase model predictability (Figs 1c(1), 2c(1); Table S2). In conclusion, the decrease in soil respiration and overall shoot mass with increasing stressor number (Figs 1a(1), 2a(1)) were not essentially driven by stressor number *per se*, but only by the sampling effects. Besides, for β‐d‐1,4‐cellobiosidase activity and legume shoot mass, adding ‘number of stressors’ improved model predictability (Figs 1c(4), S1a(5)), revealing the important roles of stressor number in affecting β‐d‐1,4‐cellobiosidase activity and legume shoot mass.

As the first study addressing plant functional group evenness under multifactorial global change, our results show that although single stressors had no effect or minimal positive effects, increasing the number of concurrent stressors continuously decreased plant functional group evenness in both low and high plant diversity systems (Fig. 2a(3)). Null models explained 33.06% of the variability in plant functional group evenness, while model *R* ^2^ reached 49.73% after adding the predictor ‘number of stressors’ (Fig. 2c(3); Table S2). Evidently, the joint effects of multiple stressors differ substantially from the sum of their individual impacts, and stressor number itself also profoundly affects plant functional group evenness. This reveals the existence of strong stressor interactions, and the unpredictability of the joint effect of multiple stressors on plant community composition (Rillig *et al*., 2019). Several prior studies have found significant effects of global change on plant evenness (Walker *et al*., 2006; Hillebrand *et al*., 2008; Malchair *et al*., 2010) and varied responses of individual species that caused the change (Kardol *et al*., 2010; Zelikova *et al*., 2014; Shi *et al*., 2015; Dorji *et al*., 2018; Bucher *et al*., 2023), which likely depends on how a stressor affects resource availability, the efficiency of resource use, and the availability of other potentially growth‐limiting resources (Kardol *et al*., 2010). Recent work reported declined species evenness in a plant community with increasing number of stressors, and our result adds to this finding by displaying a similar pattern in functional group evenness (Speißer *et al*., 2022). Decreased plant evenness may further produce some negative consequences, because it is considered an important attribute affecting ecosystem functioning (Hillebrand *et al*., 2008; Orwin *et al*., 2014).

The decline in functional group evenness was a result of the heterogeneous responses of grasses, herbs, and legumes (Fig. 3b). Although the co‐occurrence of multiple stressors had a profound effect on the overall productivity of the plant community, it was not always mirrored by changes in each functional group. With a growing number of stressors, there were only subtle variations in the shoot mass of grasses and herbs, while legumes decreased remarkably (Fig. 3b). Legumes have been reported to be more vulnerable to environmental changes such as drought, N deposition, warming, freezing, ozone, and chemical pollution compared to other functional groups (Marchiol *et al*., 1999; Calvete‐Sogo *et al*., 2016; Yang *et al*., 2021a; Rycroft & Henry, 2024). Due to the associations with rhizobia to fix atmospheric nitrogen, legumes play important roles in ecosystem functioning, and a decreased biomass of legumes may decrease N input, therefore impairing ecosystem functions (Yang *et al*., 2021a). Being initially the predominant group, grasses have become increasingly dominant as the number of stressors increased (Fig. 3a). Previous work showed reductions in soil microbial abundance, diversity, and activity with a growing number of stressors (Rillig *et al*., 2019; Yang *et al*., 2022; Liu *et al*., 2024), which is also partially reflected in our results by the decline of soil respiration and the activity of some enzymes (Fig. 1a(1),a(4),a(6)). As a result, soil nutrient availability may decrease, possibly intensifying plant competition and giving advantages to dominant species (Yang *et al*., 2021a). This may be one of the probable causes for the increasing dominance of grasses. The relative abundance of different functional groups can influence multiple soil properties according to past studies (McLaren & Turkington, 2010, 2011); thus, the heterogeneous responses we observed may produce some further effects on soil.

The effects of stressors, especially single stressors, varied under different plant diversity conditions. For the majority of response variables, adding the predictor ‘plant diversity’ significantly increased the model performance (Table S2), indicating that plant diversity could alter the effects of stressors. More importantly, whether and how co‐occurring stressors interact to affect ecosystem functioning is likely also dependent on plant diversity. We found that higher plant diversity tended to give rise to more interactions among stressors, while few interactions occurred under low plant diversity conditions. In an ecosystem with more plant species, the interspecies relationships may be more complicated due to mechanisms such as niche overlap, complementarity, and partitioning (Tylianakis *et al*., 2008; Venjakob *et al*., 2016; Godoy *et al*., 2020). These interspecies relationships can mediate the effects of multiple stressors (Thompson *et al*., 2018b), leading to a more complex response pattern at the ecosystem level. Alternatively, in low plant diversity systems, since fewer species are present, the effects of multiple stressors are likely to be less affected by interspecies relationships, and therefore be more straightforward. A possible result of the complex stressor interactions is that, under the joint impact of multiple stressors, there may be greater uncertainty in predicting the magnitude of change within the high plant diversity systems.

Plant diversity plays a fundamental role in providing a wide range of ecosystem services and maintaining ecosystem stability (Isbell *et al*., 2011). In this study, plant diversity mainly influenced soil properties and functions directly, rather than through indirect pathways mediated by plant biomass (Table S3). Although a large body of evidence suggests that ecosystems with higher plant diversity generally exhibit higher levels of functioning and stability (Isbell *et al*., 2011, 2015; Wagg *et al*., 2022), our results revealed not only positive, but also neutral and even negative effects of plant diversity, which are partially contradicting our second hypothesis. Nonetheless, nonpositive effects of plant diversity on ecosystem functioning have also been reported in previous studies (Pfisterer & Schmid, 2002; Kennedy *et al*., 2003; Firn *et al*., 2007; Fornara *et al*., 2009; Behl *et al*., 2011; Grossiord *et al*., 2014; Chen *et al*., 2017; Wu *et al*., 2018; Shovon *et al*., 2024). A possible explanation for our study is the equal number of functional groups (*n* = 3) across all treatments, as there could be functional redundancy within functional groups in the high plant diversity systems according to the redundancy hypothesis (Walker, 1992; Pillar *et al*., 2013; Kang *et al*., 2015). Functional groups integrate multiple plant traits, and therefore may better explain ecosystem functions (Thomas *et al*., 2019). For instance, leaf traits are essential in determining the ecological functions of a plant, while *P. pratense*, *L. perenne*, and *D. glomerata*, which are all cool‐season (C_3_) grasses, have been reported to have similar leaf traits such as leaf length, specific leaf area, leaf density, and leaf water content (Sugiyama, 2005). Prior research also showed similar leaf and root traits of *A. millefolium* and *D. carota* (both herbs), as well as similar root traits of *T. repens* and *M. lupulina* (both legumes) (Lozano *et al*., 2020). In some controlled environment room and field studies, functional group diversity has been shown to surpass plant species diversity in affecting biomass production, stress tolerance, microbial activity, and diversity (Spehn *et al*., 2000; Johnson *et al*., 2003; Lanta & Lepš, 2006; Komainda *et al*., 2020). Another possible reason is the relatively short duration of our experiment. Some ecosystem functions may respond positively to plant diversity change with a certain time lag, especially soil biota‐mediated functions, because positive associations between soil biota and diverse plant communities need time to develop (Eisenhauer *et al*., 2012; Vogel *et al*., 2013; Thakur *et al*., 2015; Zhang *et al*., 2023). In summary, when functional group diversity is equal, higher plant species diversity does not necessarily translate to higher levels of ecosystem functioning in multifactorial global change studies based on short‐term, small‐scale controlled experiments.

Aside from the diversified effects of plant diversity on different aspects of the plant–soil systems, we found continuing positive effects on plant functional evenness with an increasing number of stressors. This contrasts with the conclusion of earlier research showing negative relationships between plant species richness and evenness (Wilsey *et al*., 2005; Zhang *et al*., 2012; Yan *et al*., 2023). Their datasets, however, were obtained by measuring samples from natural grasslands or long‐term field experiments, while our experiment was conducted in a controlled environment room using plant–soil mesocosms, and none of them involved global change. In our case, although plant diversity does not always benefit soil functioning, it consistently helps maintain plant functional group evenness under multifactorial global change.

The effect of plant diversity was usually more remarkable when fewer stressors were applied. Regardless of whether plant diversity improved or impaired the functioning of plant–soil systems, an increasing number of stressors, as hypothesized, generally tended to diminish the effects of plant diversity. This trend was particularly conspicuous in β‐d‐1,4‐cellobiosidase activity, β‐1,4‐N‐acetyl‐glucosaminidase activity, root mass, WSA, and legume shoot mass (Figs 1b(4),b(5), 2b(2), S1b(1),b(5)). Although the effect of plant diversity was found to be robust to individually acting stressors (Craven *et al*., 2016; Eisenhauer *et al*., 2018; Cheng *et al*., 2024), our result suggests that it may be eliminated by a multifactorial stress combination. A recent study revealed weakened effects of soil microbial diversity on ecosystem functions along a gradient of increasing stressor number (Yang *et al*., 2022), and our results complement this finding by showing a similar pattern in terms of plant diversity. Increasing the number of stressors imposes a growing pressure on soil microorganisms, decreasing microbial diversity and abundance, and therefore, attenuating ecosystem functions underpinned by soil microorganisms (Yang *et al*., 2022; Liu *et al*., 2024), which may not be compensated by increasing plant diversity (Yang *et al*., 2021b). On the other hand, how plant diversity affects ecosystem functions is also largely mediated by soil microorganisms (Cappelli *et al*., 2022). As such, a decrease in microbial diversity and abundance may impair the link between plant diversity and ecosystem functions, leading to the mitigation of plant diversity effects with an increasing number of stressors.

In summary, our study indicates that a growing number of co‐acting anthropogenic stressors drive directional changes in soil properties and functions irrespective of plant diversity level. The high vulnerability of legumes compared to herbs and grasses causes a continuous decrease in plant functional group evenness as stressor number increases. Due to high‐order factor interactions, the joint effects of numerous stressors can be unexpectedly strong even if their individual effects are minimal, suggesting that increasing stressor number raises the uncertainty of predicting the changes in plant–soil systems. Plant diversity mediates the effects of single stressors, but high plant diversity does not always assure improved ecosystem functioning. In addition, when acting in concert, stressors tend to interact more to affect the plant–soil systems under high plant diversity conditions, and less under low plant diversity conditions. The concurrence of a large number of stressors tends to lead to the convergence of the low and high plant diversity systems, that is, the greater the number of stressors acting in concert, the smaller the impact of plant diversity on ecosystem functioning. These findings underline the necessity for global change research to prioritize the simultaneous incidences of multiple stressors and to pay more attention to their impacts on plant–soil systems, especially in plant communities. More importantly, plant diversity within the community should be integrated as an important variable shaping the effect of multifactorial global change. During plant diversity management in ecosystem restoration practice, the number of coexisting stressors should be taken into consideration to avoid ineffective effort. Instead of undertaking the challenge of eliminating all the existing stressors, focusing on reducing a few of them may be a more efficient strategy, yielding disproportionately positive results.

For research that simultaneously manipulates an increasing number of anthropogenic stressors and different levels of plant diversity, it remains challenging to disentangle the effects of stressor number and plant diversity. Future studies could incorporate plant monocultures and assess detailed functional traits of different plant species, while adopting systematic approaches that balance experimental complexity and feasibility, to dissect the mechanisms underlying the relationships between stressor number and the effect of plant diversity on plant–soil ecosystems. Moreover, it is also crucial to address how plant–soil ecosystems, once the plant community has been fully established and stabilized, recover towards their equilibrium following the simultaneous incidences of multiple anthropogenic stressors, and whether higher plant diversity enhances ecosystem resilience in this context.

## Competing interests

None declared.

## Author contributions

YZ, PM, HL and MB conceptualized the study and carried out the experimental setup. YZ, PM, HL, MB and MCR designed the experiments. YZ and PM analyzed the data and wrote the manuscript. MR and MCR reviewed and edited the manuscript. All authors contributed to the article and approved the final manuscript. YZ and PM contributed equally to this work.

## Disclaimer

The New Phytologist Foundation remains neutral with regard to jurisdictional claims in maps and in any institutional affiliations.

## Supporting information


**Fig. S1** Soil water stable aggregates, pH, and shoot mass of grasses, herbs, and legumes in response to different levels of anthropogenic stressors and plant diversity.
**Fig. S2** Changes in soil properties, functions, and plant community composition with increasing number of anthropogenic stressors under low and high plant diversity conditions.
**Methods S1** Rationale for the anthropogenic stressors.
**Methods S2** Detailed procedures for the measurements of response variables.

**Table S1** Significance test for the effects of anthropogenic stressor treatments.
**Table S2** Predictability comparison of the hierarchically nested models.
**Table S3** Partial least squares path modeling (PLS‐PM) revealing the direct and indirect effects of experimental treatments on plant biomass, soil properties, and functions.
Please note: Wiley is not responsible for the content or functionality of any Supporting Information supplied by the authors. Any queries (other than missing material) should be directed to the *New Phytologist* Central Office.

## Data Availability

The raw data of this manuscript are deposited in figshare under doi: 10.6084/m9.figshare.27991472.v2.
