# Supplementary material for: Concurrent anthropogenic stressors affect plant–soil systems with different plant diversity levels
Source: New Phytol. 2025 Jun 4;247(4):1897–911. doi: 10.1111/nph.70275 (PMC12267938; doi:10.1111/nph.70275)
Supplement: Supplementary file 1 — Fig. S1 Soil water stable aggregates, pH, and shoot mass of grasses, herbs, and legumes in response to different levels of anthropogenic stressors and plant diversity. Fig. S2 Changes in soil properties, functions, and plant community composition with increasing number of anthropogenic stressors under low and high plant diversity conditions. Methods S1 Rationale for the anthropogenic stressors. Methods S2 Detailed procedures for the measurements of response variables. [file NPH-247-1897-s002.pdf]

New Phytologist

***New Phytologist* Supporting Information**

Article title: Concurrent anthropogenic stressors affect plant-soil systems with different plant diversity levels

Authors: Yanjie Zhu, Peter Meidl, Huiying Li, Mohan Bi, Masahiro Ryo, Matthias C. Rillig

Article acceptance date: 16 May 2025

The following Supporting Figures and Methods are available for this article:

Fig. S1 Soil water stable aggregates, pH and shoot mass of grasses, herbs and legumes in response to different levels of anthropogenic stressors and plant diversity.

Fig. S2 Changes in soil properties, functions and plant community composition with increasing number of anthropogenic stressors under low and high plant diversity conditions.

Methods S1 The rationale for the anthropogenic stressors.

Methods S2 The detailed procedures for the measurements of response variables.

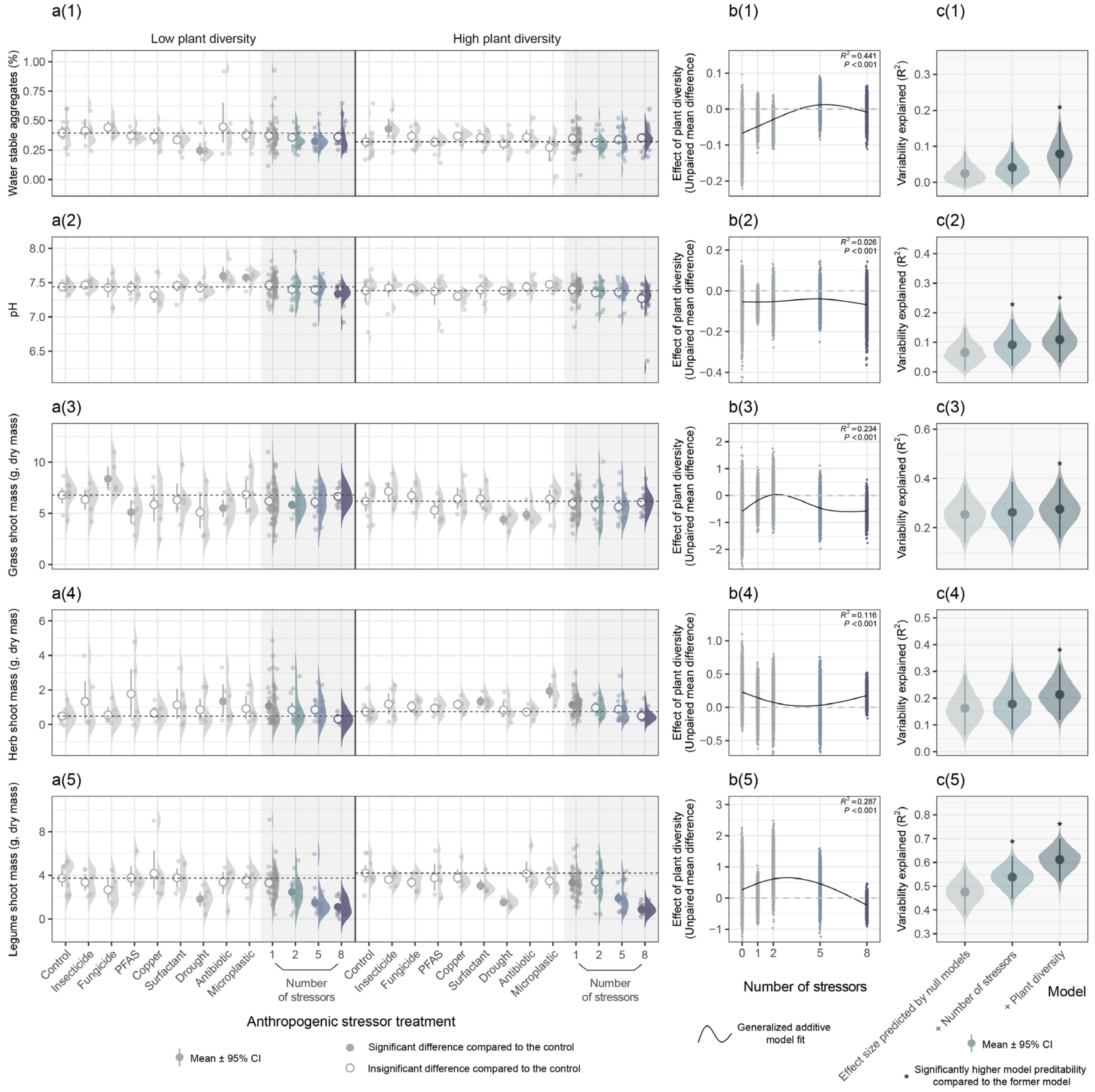

Fig. S1 Soil water stable aggregates, pH and shoot mass of grasses, herbs and legumes in response to different levels of anthropogenic stressors and plant diversity. [a(1)-a(5)] Raw data distribution, mean and confidence interval (CI) of each response variable in treatments with single and multiple (2, 5 and 8) stressors under low and high plant diversity conditions. The filled circle represents a significant difference ($p<0.05$) compared to the control, while the empty circle represents an insignificant difference ($p>0.05$) compared to the control. The p-values were calculated based on bootstrap resampling with 5000 iterations (Supporting Information Table S1). The horizontal dashed line represents the mean value of the control. [b(1)-b(5)] Correlations between the effect of plant diversity (unpaired mean difference between the low and high plant diversity treatments) on each response variable and the number of anthropogenic stressors. [c(1)-c(5)] Variability of response variable

explained by generalized additive models. The baseline model using effect size predicted by null models represents the contribution of factor identity, while added predictors represent the contributions of stressor number and plant diversity. The model comparisons were performed using ANOVA tests (Supporting Information Table S2).

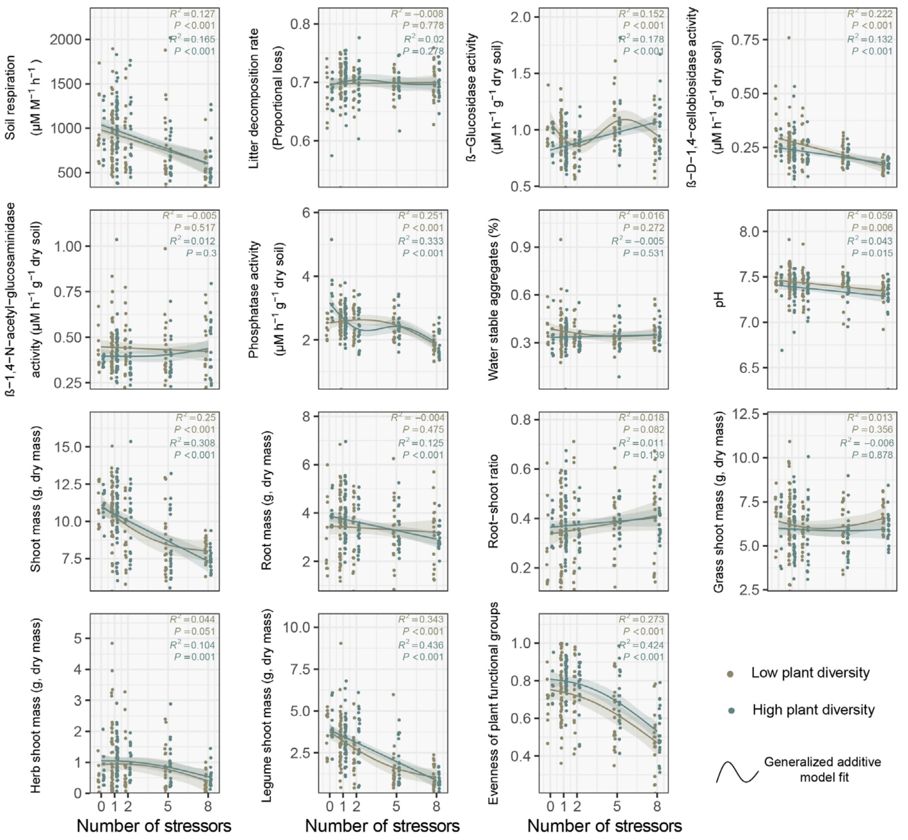

Fig. S2 Changes in soil properties, functions and plant community composition with increasing number of anthropogenic stressors under low and high plant diversity conditions.

Methods S1 The rationale for the anthropogenic stressors.

We here present the rationale for the 8 stressors:

1) *Insecticide*. We used imidacloprid (PESTANAL ® analytical standard, Sigma-Aldrich, MO, USA), which is one of the most widely applied insecticides globally (Rillig *et al.*, 2019). Its concentration in German and UK agricultural soils ranged from 1.6 up 50 ng g^{-1} (Wood & Goulson, 2017), we thus used the dose of 50 μg kg^{-1} in our experiment.
2) *Fungicide*. We used carbendazim (PESTANAL ® analytical standard, Sigma-Aldrich, MO, USA), a fungicide that is commonly applied on humans, animals and plants in agricultural fields (Rillig *et al.*, 2019). It has been used previously in soil research, with a recommended field application dose of about 6.0 mg kg^{-1} and 20 and 40-fold this concentration (Lang & Cai, 2009). Other studies have used 1 mg kg^{-1} to 100 mg kg^{-1} (Wang *et al.*, 2016). We used 6.0 mg kg^{-1} in our experiment (Rillig *et al.*, 2019).
3) *Antibiotic*. We used oxytetracycline (added as oxytetracycline-dihydrate; Sigma-Aldrich, MO, USA), an antibiotic applied on humans and animals, which will be introduced to soil environment by processes such as the application of manure or slurry (Kemper, 2008). It has been detected in soil at a concentration of 305.0 ng kg^{-1}, and was shown to persist and accumulate in soil (Kemper, 2008). We used 3.0 mg kg^{-1} in our experiment to simulate a temporary hotspot, resulting for example from a fresh deposition of manure on an agricultural field (Rillig *et al.*, 2019).
4) *Heavy metal*. We used copper (added as copper(ii)-sulphate-pentahydrate; BioChemica. AppliChem GmbH, Darmstadt, Germany), an important heavy metal pollutant in European soils (Ballabio *et al.*, 2018). Copper concentrations in German soil range typically from 2 to 50 mg kg^{-1}, and we used 100 mg kg^{-1} in our experiment to simulate a hotspot of copper contamination, which can occur in areas such as mining sites (Rillig *et al.*, 2019).
5) *Perfluoroalkyl and polyfluoroalkyl substances (PFAS)*. Perfluoroalkyl and polyfluoroalkyl substances (PFAS) as a highly diverse class of pollutant are of great concern due to their high persistence and widespread distribution in the environment (Brusseau *et al.*, 2020). Their concentration in contaminated sites can be up to several hundred mg kg^{-1} (Brusseau *et al.*, 2020). We here used perfluorooctanoic acid (PFOA; Strem Chemicals Inc., Bellevue, WA, USA) with a concentration of 1 mg kg^{-1}, which is of environment relevance for agricultural soils in Germany (Brusseau *et al.*, 2020; Xu *et al.*, 2022; Xu *et al.*, 2023).
6) *Surfactant*. Surfactants are widely used in household and industrial products. After use, they can enter soil through sludge disposal (Ying, 2006). We here used sodium dodecylbenzenesulfonate (SDBS; Sigma-Aldrich, MO, USA) in our experiment and used an environmentally relevant concentration of 16 mg kg^{-1} (Ying, 2006).
7) *Microplastics*. We used tire wear particles (TWP; KURZ-karkassenhandel GmbH, Wendlingen, Germany; size range, 1-2 mm), since TWP is a major source of microplastics in the environment (Sommer *et al.*, 2018), and has been recognized as an emerging threat to soil health globally recent years (Ding *et al.*, 2022). The concentrations of TWP

in roadside soil in Germany range from 0.4 g kg^{-1} to 158.0 g kg^{-1} (Baensch-Baltruschat *et al.*, 2021), we thus used a concentration of 1 g kg^{-1} in our experiment.

8) *Drought*. As a main factor of global environmental change, drought has strong effects on soils (Metze *et al.*, 2023). It was predicted that drought events will occur more frequently and severely in many regions in the future (Dai, 2013; Hari *et al.*, 2020). We here represented drought by reducing the soil water content to 30% water holding capacity (WHC), while the water content of 60% WHC was used for the soils without drought treatment. The drought treatment didn't start until the 6th week, because the plant seedlings were too small in the early stage and could die easily under drought stress.

Methods S2 The detailed procedures for the measurements of response variables.

1) *Decomposition rate*. Litterbags with a size of ca. 1.5 × 3.0 cm were manually made using a nylon mesh (pore size, 38 μm), with each of the bag containing 350 mg green tea (Lipton green tea, Sencha Exclusive Selection, Unilever Food Solutions, London, UK). The litterbags were placed in the center of the soils, and their position was kept consistent across all the pots. At the final harvest, the litterbags were collected, oven-dried and weighed, and the percentage of weight loss was used as an indicator of soil decomposition rate (Xie, 2020).
2) *Soil respiration*. Thirty grams of fresh soil was placed in a 50 mL falcon tube equipped with a rubber stopper on the lid. The tubes were flushed with CO_2-free air for 5 min to eliminate background CO_2 and then incubated for 4 h at 20 °C. Then, 1 mL of air in the headspace of the tube was extracted using a syringe and injected into an infrared gas analyzer (LI-6400XT, LI-COR Inc., Bad Homburg, Germany) to determine CO_2 concentration. Soil respiration was indicated by the CO_2 concentration produced per hour (μM M^{-1} h^{-1}) (Rillig *et al.*, 2019).
3) *Extracellular enzyme activities*. Five grams of fresh soil was placed in a 50 mL falcon tube, and 10 mL of 50 mM acetate buffer (pH, 5.0−5.5) was added. The tubes were vortexed for 5 secs, and each soil slurry (150 μL) into each well (6 wells per sample) in the 96-well plate. Then, 150 μL of each substrate was added: *p*NP-β-D-glucopyranoside (Sigma no. N7006), *p*NP-β-D-cellobioside (Sigma no. N5759), *p*NP-N-acetyl-β-D-glucosaminide (Sigma no. N9376), *p*NP- phosphate disodium salt hexahydrate (Sigma no. 71,768). After 2 h (β-glucosidase and phosphatase) and 4 h (β-D-1,4-cellobiosidase and β-1,4-N-acetyl-glucosaminidase) incubations at 20°C under dark conditions, each plate was centrifuged at 3,000 rpm for 5 min, and 100 μL of supernatant was transferred into a new plate and mixed with 200 μL of 0.1 M NaOH solution. Absorbances were determined at 410 nm using a microplate reader (Benchmark Plus, BioRad Laboratories GmbH, Hercules, CA, USA), and each enzyme activity was calculated as μmol p-nitrophenol g soil dry $mass^{-1}$ h^{-1} (Jackson *et al.*, 2013).
4) *Water stable aggregates*. We followed the wet sieving method of Kemper and Rosenau (Kemper & Rosenau, 1986) with a slight modification. Four grams of air-dried soil was put in a small sieve with a mesh size of 0.25 mm, re-wetted by capillarity with deionized water and inserted into a sieving machine (Agrisearch Equipment, Royal Eijkelkamp B.V., Giesbeek, Netherlands) to be wet-sieved for 3 min. The soil left on the sieve (fraction 1: stable soil aggregates and coarse matter) was dried at 60°C and weighed, and then crushed manually in the wet sieve to obtain the coarse matter (fraction 2). Calculations of the percentage of WSA were according to: WSA (%) = (fraction 1 – fraction 2)/(4.0 – fraction 2) × 100% (Xu *et al.*, 2022).
5) *pH*. Five grams of air-dried soil was put in a 50 mL falcon tube, and 12.5 mL 0.01 M $CaCl_2$ solution was added. The tube was shaken in 250 rpm for 30 min and then centrifuged in 4600 rpm for 10 min. The supernatant was filtered, and the pH was determined by a pH-meter (Hanna Instruments GmbH, Smithfield, USA).

References

Baensch-Baltruschat B, Kocher B, Kochleus C, Stock F, Reifferscheid G. 2021. Tyre and road wear particles - A calculation of generation, transport and release to water and soil with special regard to German roads. *Science of the Total Environment* **752**: 141939.

Ballabio C, Panagos P, Lugato E, Huang JH, Orgiazzi A, Jones A, Fernández-Ugalde O, Borrelli P, Montanarella L. 2018. Copper distribution in European topsoils: An assessment based on LUCAS soil survey. *Science of the Total Environment* **636**: 282-298.

Brusseau ML, Anderson RH, Guo B. 2020. PFAS concentrations in soils: Background levels versus contaminated sites. *Science of the Total Environment* **740**: 140017.

Dai AG. 2013. Increasing drought under global warming in observations and models. *Nature Climate Change* **3**(1): 52-58.

Ding J, Lv M, Zhu D, Leifheit EF, Chen QL, Wang YQ, Chen LX, Rillig MC, Zhu YG. 2022. Tire wear particles: An emerging threat to soil health. *Critical Reviews in Environmental Science and Technology* **53**: 239-257.

Hari V, Rakovec O, Markonis Y, Hanel M, Kumar R. 2020. Increased future occurrences of the exceptional 2018-2019 Central European drought under global warming. *Scientific Reports* **10**(1).

Jackson CR, Tyler HL, Millar JJ. 2013. Determination of Microbial Extracellular Enzyme Activity in Waters, Soils, and Sediments using High Throughput Microplate Assays. *Jove-Journal of Visualized Experiments*(80).

Kemper N. 2008. Veterinary antibiotics in the aquatic and terrestrial environment. *Ecological Indicators* **8**(1): 1-13.

Kemper W, Rosenau R. 1986. Aggregate stability and size distribution. In Methods of soil analysis: Part 1 physical and mineralogical methods. *Soil Science Society of America Journal*: 425-442.

Lang M, Cai ZC. 2009. Effects of chlorothalonil and carbendazim on nitrification and denitrification in soils. *Journal of Environmental Sciences* **21**(4): 458-467.

Metze D, Schnecker J, Canarini A, Fuchslueger L, Koch BJ, Stone BW, Hungate BA, Hausmann B, Schmidt H, Schaumberger A, et al. 2023. Microbial growth under drought is confined to distinct taxa and modified by potential future climate conditions. *Nature Communications* **14**(1): 5895.

Rillig MC, Ryo M, Lehmann A, Aguilar-Trigueros CA, Buchert S, Wulf A, Iwasaki A, Roy J, Yang GW. 2019. The role of multiple global change factors in driving soil functions and microbial biodiversity. *Science* **366**(6467): 886-890.

Sommer F, Dietze V, Baum A, Sauer J, Gilge S, Maschowski C, Giere R. 2018. Tire abrasion as a major source of microplastics in the environment. *Aerosol and Air Quality Research* **18**(8): 2014-2028.

Wang CX, Wang FF, Zhang QM, Liang WX. 2016. Individual and combined effects of tebuconazole and carbendazim on soil microbial activity. *European Journal of Soil Biology* **72**: 6-13.

Wood TJ, Goulson D. 2017. The environmental risks of neonicotinoid pesticides: a review of the evidence post 2013. *Environmental Science and Pollution Research* **24**(21): 17285-17325.

Xie YJ. 2020. A meta-analysis of critique of litterbag method used in examining decomposition of leaf litters. *Journal of Soils and Sediments* **20**(4): 1881-1886.

Xu BL, Alizray R, Lammel DR, Riedel S, Rillig MC. 2022. Concentration-dependent response of soil parameters and functions to trifluoroacetic acid. *European Journal of Soil Science* **73**(4).

Xu BL, Yang GW, Lehmann A, Riedel S, Rillig MC. 2023. Effects of perfluoroalkyl and polyfluoroalkyl substances (PFAS) on soil structure and function. *Soil Ecology Letters* **5**(1): 108-117.

Ying GG. 2006. Fate, behavior and effects of surfactants and their degradation products in the environment. *Environment International* **32**(3): 417-431.
